# A novel Alzheimer’s disease prognostic signature: identification and analysis of glutamine metabolism genes in immunogenicity and immunotherapy efficacy

**DOI:** 10.1038/s41598-023-33277-x

**Published:** 2023-04-27

**Authors:** Zixuan Wu, Ping Liu, Baisheng Huang, Sisi Deng, Zhenyan Song, Xindi Huang, Jing Yang, Shaowu Cheng

**Affiliations:** 1 Hunan University of Chinese Medicine, Changsha, 410128 China; 2 Guangzhou University of Chinese Medicine, Guangzhou, 510120 China

**Keywords:** Computational biology and bioinformatics, Drug discovery, Immunology, Neuroscience, Biomarkers, Diseases

## Abstract

Alzheimer’s disease (AD) is characterized as a distinct onset and progression of cognitive and functional decline associated with age, as well as a specific neuropathology. It has been discovered that glutamine (Gln) metabolism plays a crucial role in cancer. However, a full investigation of its role in Alzheimer’s disease is still missing. This study intended to find and confirm potential Gln-related genes associated with AD using bioinformatics analysis. The discovery of GlnMgs was made possible by the intersection of the WGCNA test and 26 Gln-metabolism genes (GlnMgs). GlnMgs’ putative biological functions and pathways were identified using GSVA. The LASSO method was then used to identify the hub genes as well as the diagnostic efficiency of the four GlnMgs in identifying AD. The association between hub GlnMgs and clinical characteristics was also studied. Finally, the GSE63060 was utilized to confirm the levels of expression of the four GlnMgs. Four GlnMgs were discovered (ATP5H, NDUFAB1, PFN2, and SPHKAP). For biological function analysis, cell fate specification, atrioventricular canal development, and neuron fate specification were emphasized. The diagnostic ability of the four GlnMgs in differentiating AD exhibited a good value. This study discovered four GlnMgs that are linked to AD. They shed light on potential new biomarkers for AD and tracking its progression.

## Introduction

Alzheimer’s disease (AD) is often regarded as one of the primary causes of dementia and frailty. The signs of the illness begin with mild memory issues and proceed to cognitive impairment, dysfunctions in complex daily tasks, and several other domains of cognition. By the time AD is clinically recognized, neuronal loss and neuropathologic abnormalities have devel- oped in several brain locationst^1,2^. AD is a degenerative and irreversible brain disease that impairs memory, cognition, and, eventually, the ability to perform even the most basic activities. Injury appears to begin in the hippocampus and entorhinal cortex, two areas of the brain critical for memory formation^3^. Additional brain regions are harmed as more neurons die, and brain tissue is substantially reduced in the latter stages of AD. While numerous variables, like as genetics and lifestyle, impact a person’s risk of acquiring AD, age is by far the most important^4^. The condition is rare before the age of 65, and the recurrence grows in subsequent decades, with a 24–33% probability of having the disease by the age of 85^5^.Given the pessimistic projections for the AD population and its associated socioeconomic costs between 2030 and 2050, scientific and clinical research in the field of AD is currently focusing on the early detection of the transitional phase between normal aging, moderate cognitive impairment, and dementia^6,7^. Throughout the preceding three decades, much has been learnt about the molecular basis of the condition, stressing the potential for developing biomarkers for diagnosis, risk assessment, clinical trials, therapeutic targeting, and discovering novel pharmaceutical targets^8,9^.

AD is a complex disease that is unlikely to be successfully treated with a single medication or other intervention. Modern pharmacotherapeutic strategies are focused on supporting patients in maintaining mental capacities, regulating behavioral manifestations, and delaying development, hence decreasing the appearance of sickness symptoms^10,11^. All currently known treatments work by altering the levels of particular neurotransmitters in the brain, principally acetylcholine (ACh) and glutamate. Although this helps with symptoms, it is not a total cure for the condition^12^. With the growth of bioinformatics, a lot of evidence-based evidence has been gathered. Computation and prediction based on relevant information can give certain alternative suggestions for future clinical diagnosis and therapy and drug research.

Because glutamine (Gln) is the most prevalent amino acid in circulation, cultured tumor cells use it quickly. Gln is commonly used in cellular aerobic glycolysis to maintain TCA flux or as a source of citrate for lipid synthesis in reductive carboxylation^13^. Furthermore, glutaminolysis enhances proliferative cell survival by decreasing oxidative stress and preserving the integrity of the mitochondrial membrane. Gln serves as an energy source for both tumor and immunological cells^14^. However, it appears that inflammatory antitumor immune cells, particularly macrophages, do not rely on or even reject Gln metabolism. M2 macrophages are more dependent on Gln than naive macrophages, but decreased Gln metabolism can generate pro-inflammatory M1 macrophages^15^. As a result, Gln metabolism may be a target for converting tumor-associated macrophages from M2 to M1, hence increasing the anti-tumor inflammatory immune response.

Furthermore, Gln metabolism is important in Th1 cell differentiation and effector T cell activation. These data imply that inhibiting Gln metabolism may be able to restructure TME and boost immunotherapy effectiveness. Some pattern recognition receptors in AD can form huge multiprotein complexes known as inflammasomes. When inflammasomes combine, they create membrane holes and process proinflammatory cytokines, resulting in pyroptosis, a kind of inflammatory cell death^16^. Innate immune signaling and inflammasome activation are important preventive mechanisms against AD^17^. Their activation, however, must be strictly managed, since excessive activation can cause neuroinflammation and brain injury. Potential treatment methods for AD have included balancing the host’s innate immune response^18^. Although targeting Gln metabolism in conjunction with immunotherapy is incredibly promising in AD, the landscape of Gln metabolism in tumor microenvironment (TME) is yet unknown. As a result, we conducted this work to conduct a comprehensive review of GlnMgs and immunotherapy in AD.

**Figure 1:**
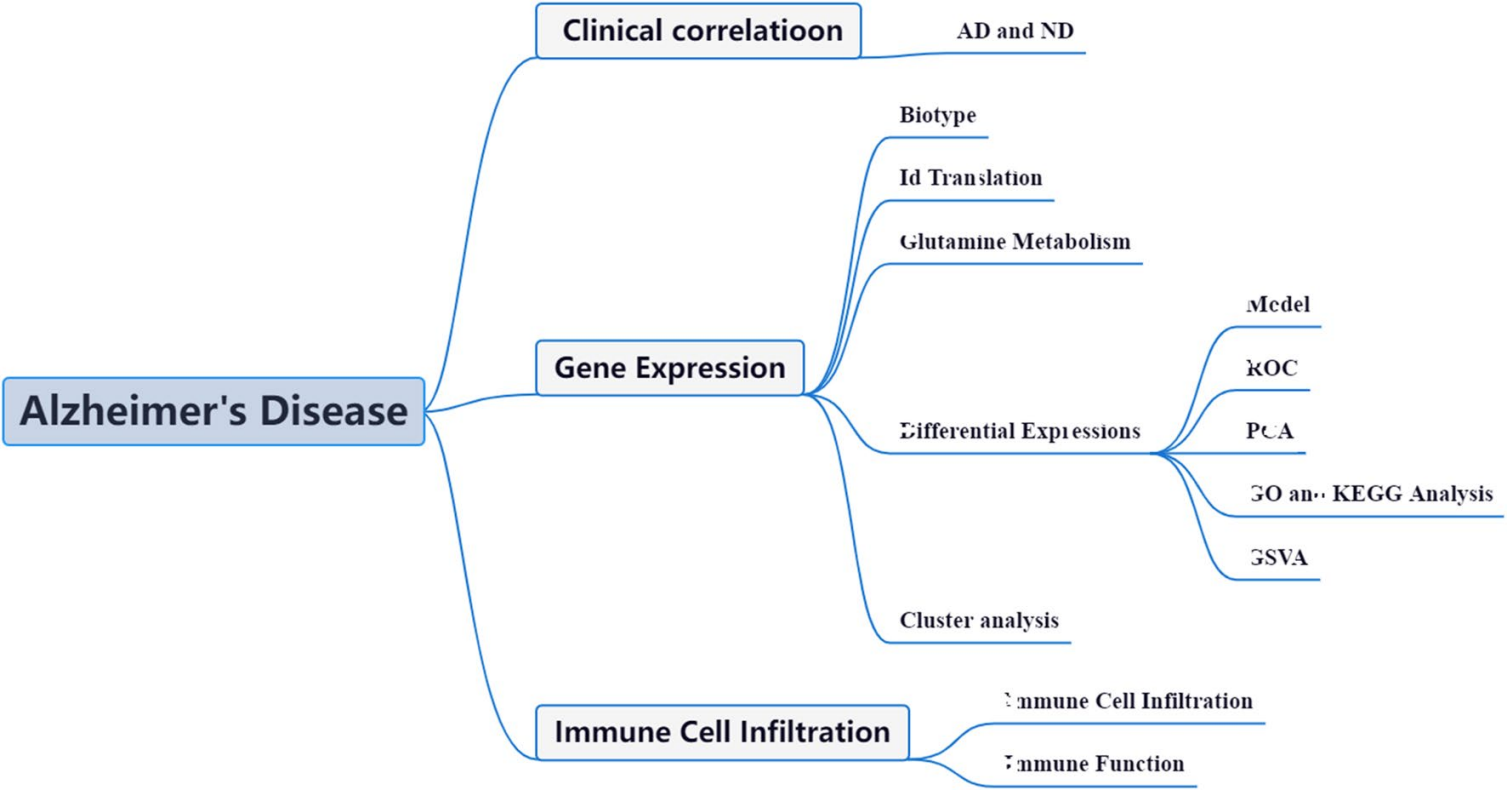
Framework. The data of AD patients were obtained from GEO databases, and then the GlnMgs were matched to carry out difference analysis and risk model construction, respectively.a˘GSE132903 was used as the main body and GSE63060 was used to verify the model with good grouping, and GlnMgs related to the prognosis of AD patients were obtained.a˘Then, GO, KEGG and GSEA analyses were performed with multiple databases to obtain the functions related to GlnMgs.a˘Last, the immune cells, function and RNA changesa˘were analyzed.

In biological research, gene expression analysis is becoming increasingly significant. The Accelerating Medicines Partnership- AD program’s availability of high-throughput transcriptome sequencing data and clinical annotation allows us to investigate the altered transcriptional and related molecular pathways implicated in AD. Several research have used gene expression information acquired from the Gene Expression Omnibus (GEO) to investigate the molecular pathways involved in the development of AD^19,20^. The results of these bioinformatics analyses provide intriguing insights for understanding the pathophysiology and processes of AD from several perspectives. However, no study has used bioinformatics to determine whether GlnMgs are im- portant for AD development. As a result, the goal of this work was to examine the AD-related GEO via the lens of the GlnMgs (Fig. 1).

### DEG identification and principal component analysis

**Figure 2:**
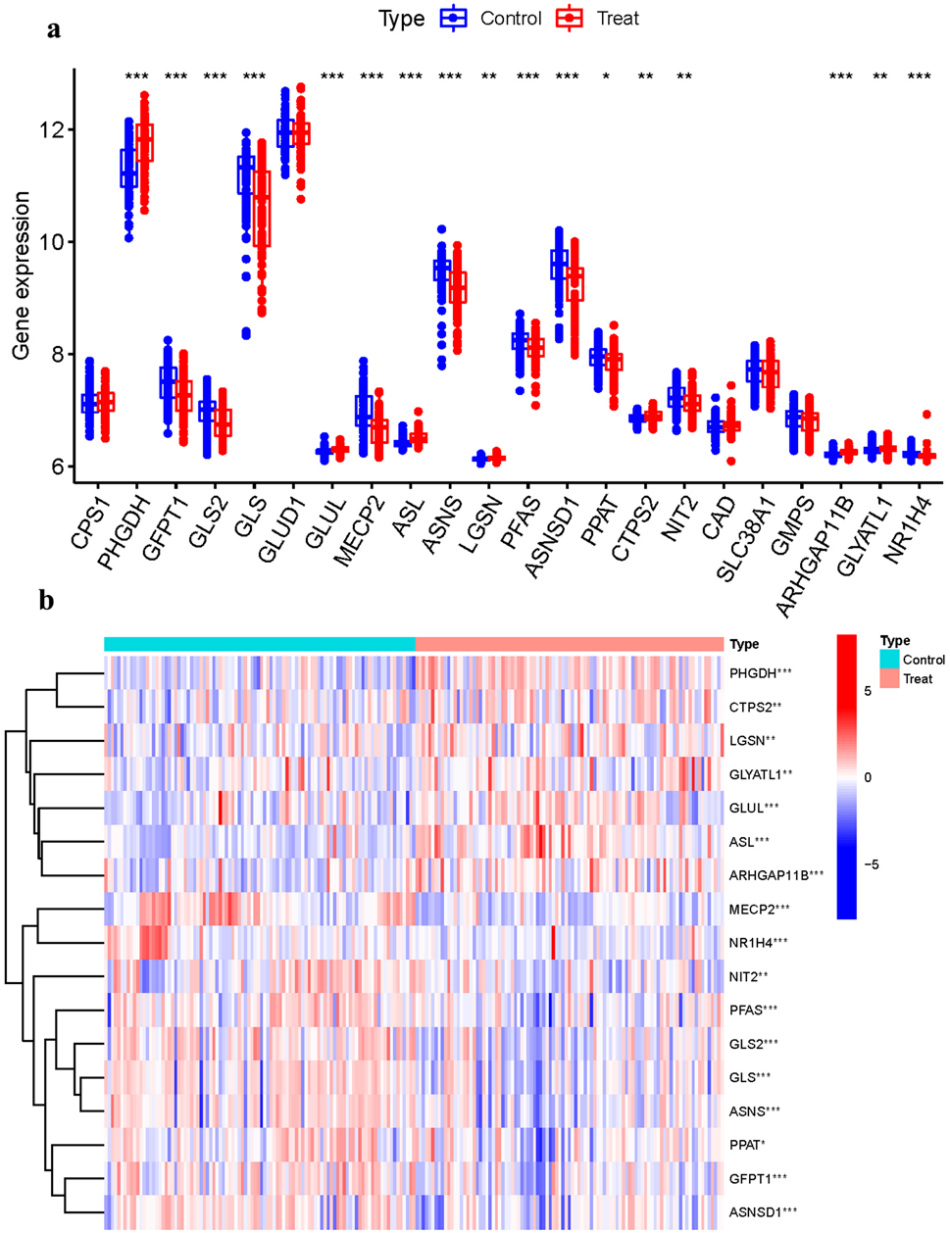
Principal component analysis. (**a**) GlnMgs. (**b**) Expression of GlnMgs in clusters.

Among the 26 GlnMgs, all were significantly different except for CPS1, GLUD1, CAD, SLC38A1,GMPS (Fig. 2a). Some genes cluster in the treat group and some in the control group. Treat: PHGDH, CTPS2, LGSN, GLYATL1, GLUL, ASL, ARHGAP11B. Control: MECP2, NR1H4, NIT2, PFAS, GLS2, GLS, ASNS, PPAT, GFPT1, ASNSD1 (Fig. 2b) (Table S2).

### Expression of GlnMgs

**Figure 3:**
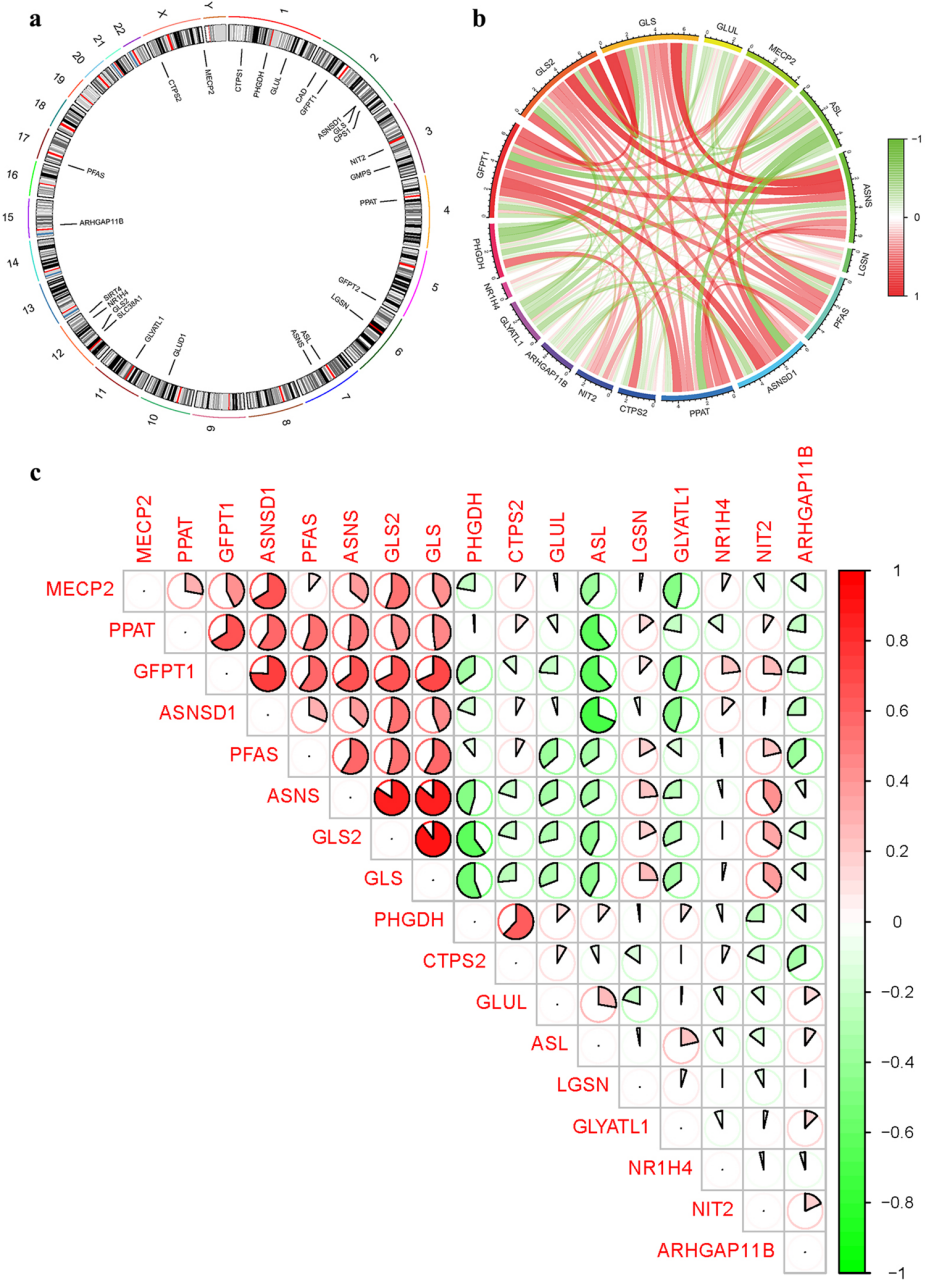
Expression of GlnMgs. (**a**) Expression of GlnMgs on sequences. (**b**,**c**) The correlation between GlnMgs and related genes.

We calculated the chromosomal positions of GlnMgs and visualized them in circles (Fig. 3a) (Table S3).a˘Then, in order to clarify the expression of these genes, we conducted correlation analysis of these genes (Fig. 3b,c).

### Immune cells

**Figure 4:**
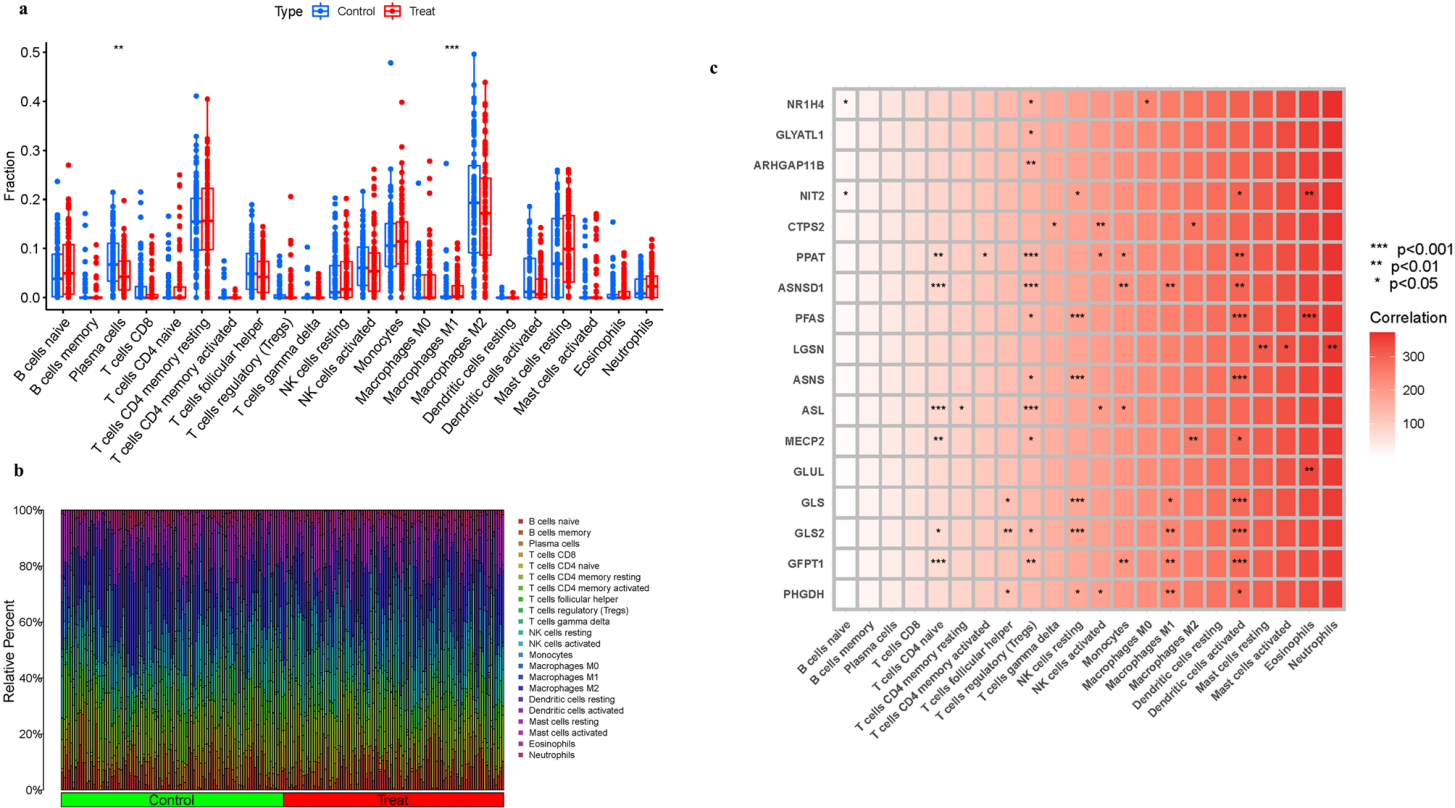
Expression of Immune cells. (**a**,**b**) Expression of immune cells in different clusters. (**c**) Correlation between GlnMgs and immune cells.

The immune environment is extremely essential in the onset and progression of AD. CIBERSORT was used to examine the immune cell components in adipose tissue. We built barplot and corplot to show the results of immune cells (Fig. 4a,b). Then, in order to clarify the expression of these genes, we conducted correlation analysis of these genes and immune cells (Fig. 4c).

### Cluster analysis

**Figure 5:**
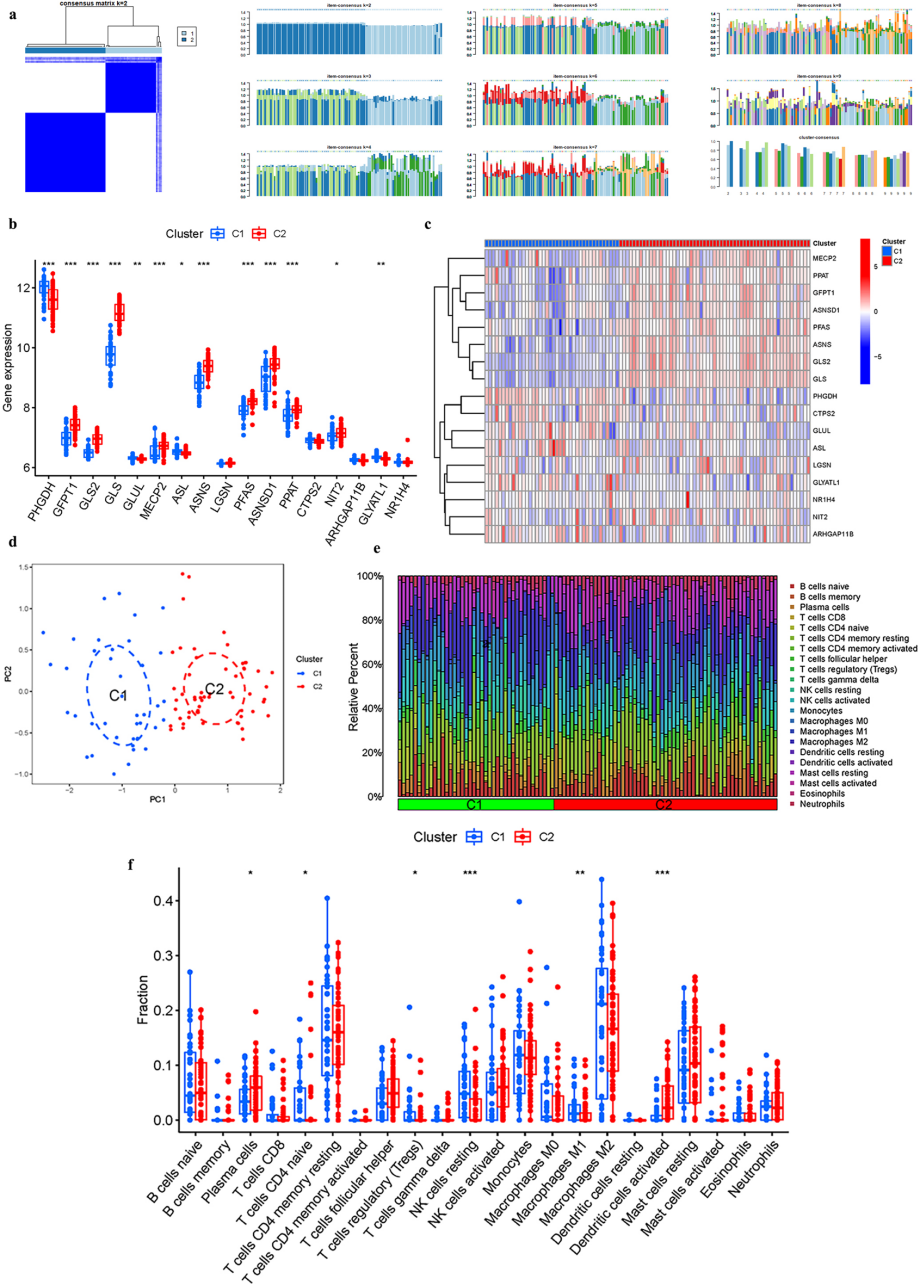
Cluster analysis. (**a**) Consensus clustering matrix. (**b**,**c**) Expression of the GlnMgs in different clusters. (**d**) PCA. (**e**,**f**) Immune cell infiltration of different clusters.

When the clustering variable (k) was set to 2, the intragroup correlations were the strongest and the intergroup correlations were the smallest, indicating that AD patients could be divided into two groups based on GlnMgs (Fig. 5a). Based on this cluster, we also discussed the expression of the GlnMgs in different clusters. CTPS2, ARHGAP11B, and NR1H4 were not significantly different between the two groups (Fig. 5b,c). According to the PCA results, patients with varying risks were divided into two groups (Fig. 5d). Based on the previous results, we also analyzed the results of immune cell infiltration according to different clusters (Fig. 5e,f).

### Analysis of functional enrichments

**Figure 6:**
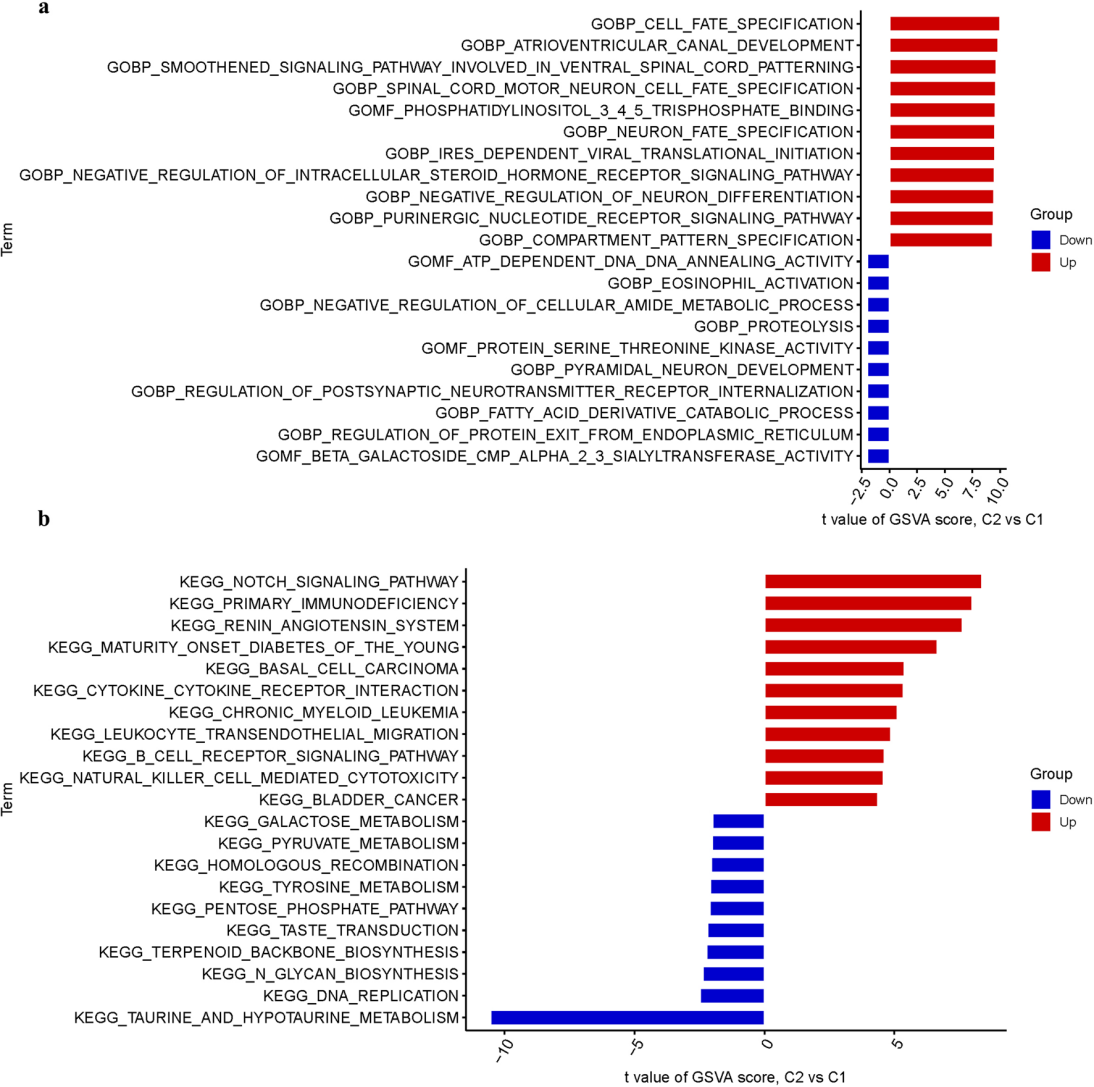
Enrichment analysis for DEGs. (**a**) GO. (**b**) KEGG. (**a**) Barplot graph for GO enrichment (the longer bar means the more genes enriched; q-value: the adjusted p-value). (**b**) Barplot graph for KEGG pathways (the longer bar means the more genes enriched).

The GlnMgs were used to conduct the enrichment analysis. The MF mainly involves atp dependent dna dna annealing activity, beta galactoside cmp alpha-2-3-sialyltransferase activity, gomf phosphatidylinositol-3-4-5-trisphosphate binding. The BP mainly involves cell fate specification, atrioventricular canal development, neuron fate specification (Fig. 6a). The pathways analysis showed that the notch signaling pathway, primary immunodeficiency, renin angiotensin system were enriched (Fig. 6b).

### Building a co-expression network and module detection

**Figure 7:**
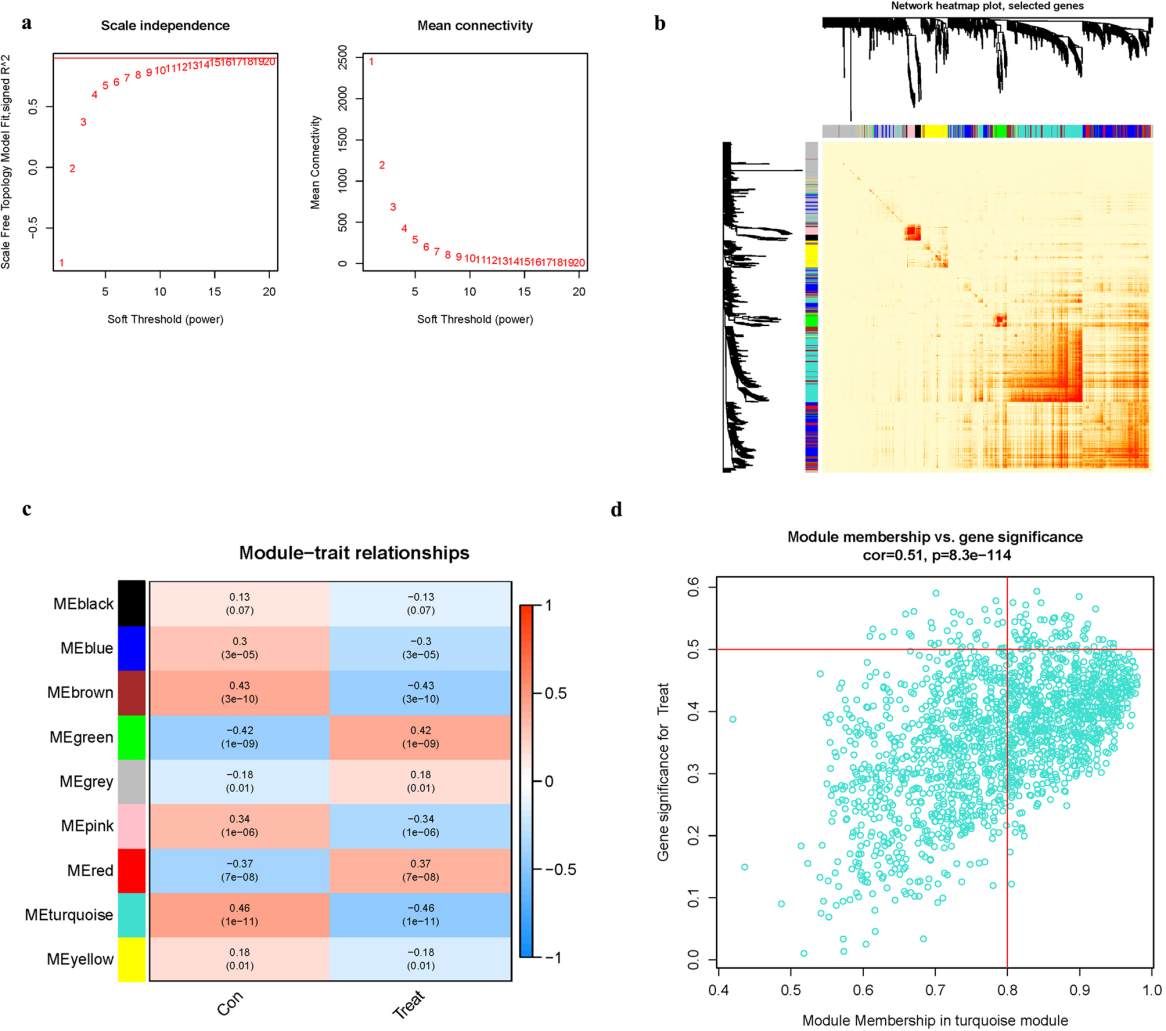
Co-expression module construction. (**a**) Soft threshold power mean connection and scale-free fitting index anal- ysis. (**b**) Clustering of dendrograms According to dynamic tree cutting, the genes were sorted into distinct modules using hierarchical clustering with a threshold of 0.25. Each color represents a separate module. (**c**) Heatmap of correlations between module eigengenes and clinical characteristics. (**d**) Gene scatterplot in the turquoise module.

To establish an approximation scale-free topology for the network, a soft-thresholding power was applied (Fig. 7a). The genes with the highest variance were grouped and integrated into nine co-expression modules (Fig. 7b). The relationship between module eigengene and clinical characteristics was investigated using Pearson’s correlation analysis (Fig. 7c). The turquoise module was shown to be highly connected with the “Group” attribute (i.e. AD and ND) and to have the greatest association (Fig. 7d) (Table S4).

### Clustering co-expression network construction and module detection

**Figure 8:**
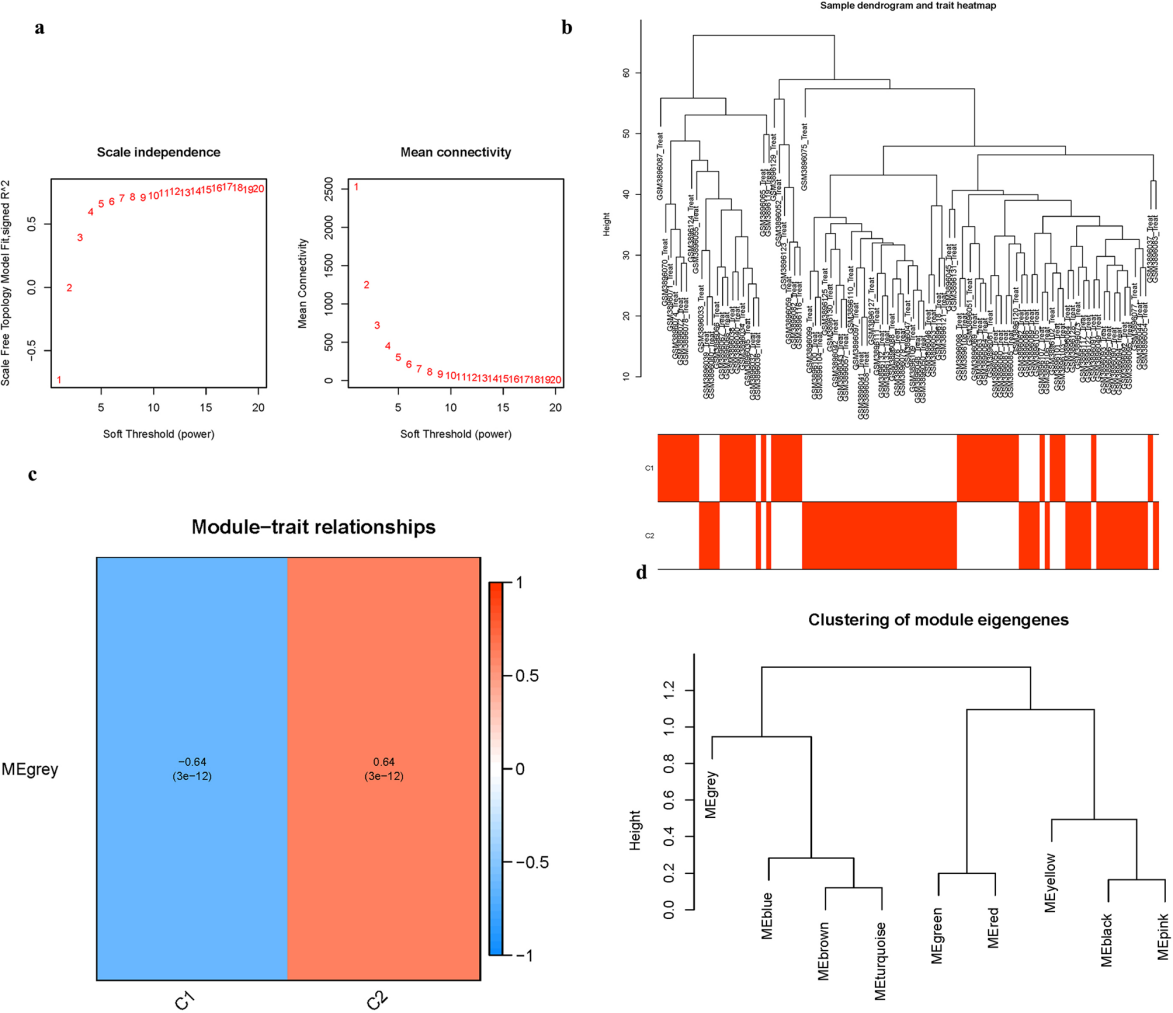
Cluster construction of co-expression modules (**a**) Soft threshold power mean connection and scale-free fitting index analysis. (**b**) Dendrogram clustering (**c**) Heatmap of correlations between module eigengenes and clinical characteristics. (**d**) Gene scatterplot in the grey module.

To establish an approximation scale-free topology for the network, a soft-thresholding power was applied (Fig. 8a). The co-expression modules were formed by clustering the variance genes (Fig. 8b). Pearson’s correlation analysis was used to investigate the relationship between module eigengene and clinical characteristics (Fig. 8c). The module was shown to be strongly linked with the “Group” characteristic (i.e. AD and ND) and to have the greatest association (Fig. 8d) (Table S5).

### Developing a model for least absolute shrinkage and operator selection

**Figure 9:**
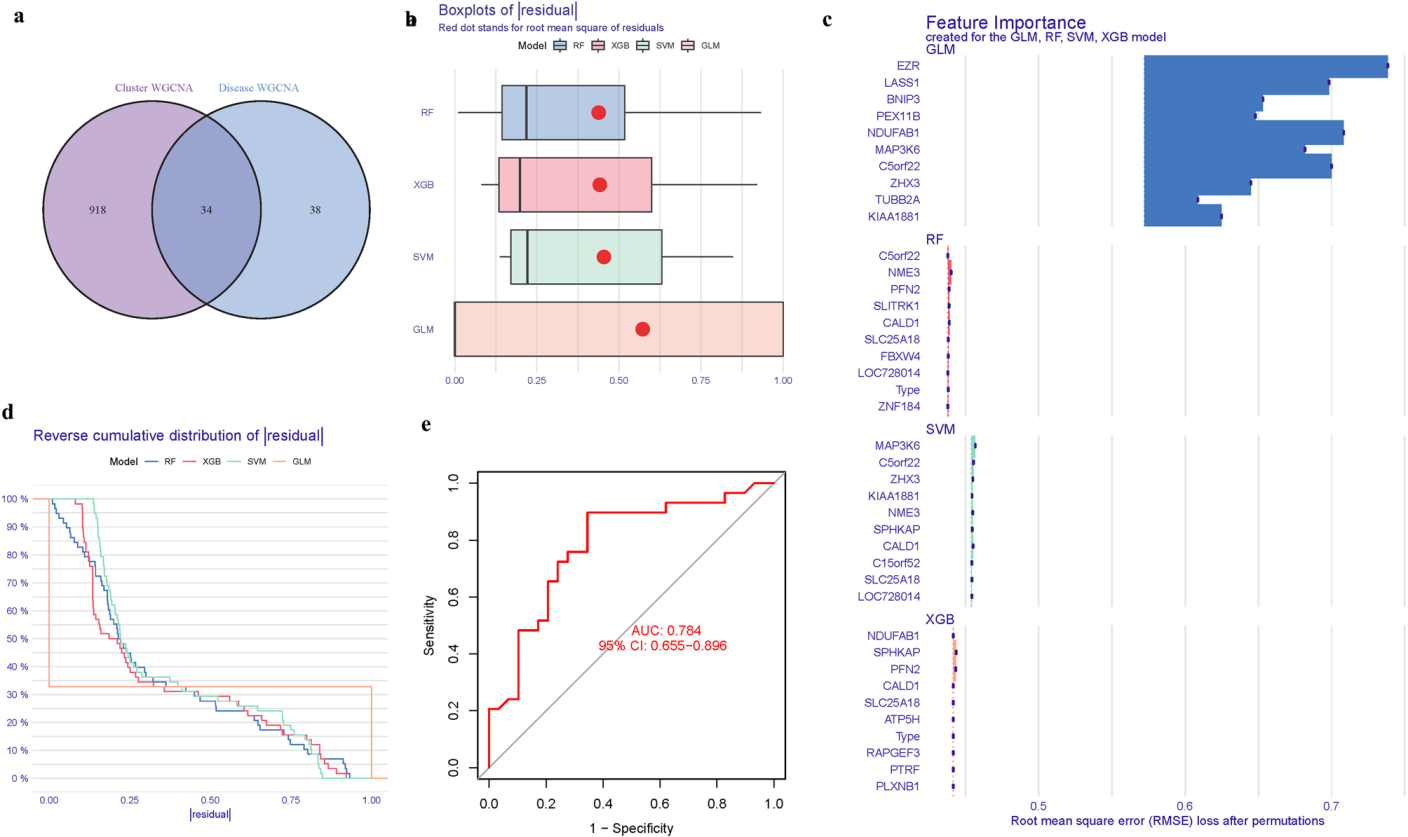
(**a**) Identification of GlnMgs with a venn diagram. (**b**,**c**) Residuala˘expression patterns. (**d**) AUC of train group. (**e**) AUC of test group.

DEGs, grey module genes (WGCNA), and GlnMgs overlapping as well. A total of 34 genes were crossed (Fig. 9a) (Table S6). The Boxplots depicted the residual expression patterns of these genes in AD (Fig. 9b).a˘There are some differences in the proportions of the four different modes (Fig. 9c). As seen in Fig. 6e, the GlnMgs’ diagnostic capacity in distinguishing AD from control samples revealed a satisfactory diagnostic value, with an Areas under the curve (AUC)a˘of RF: 0.784, SVM: 0.759, XGB: 0.788, and GLM: 0.666 (Fig. 9d). An AUC of 0.784 (95% CI 0.655–0.896) in GSE132903, an AUC of 0.815 (95% CI 0.7340.895) in GSE63060 (Fig. 9e) (Table S7).

### Drug-gene interactions

The hub gene in the XGB model predicted three drugs. These include ME-344, METFORMIN HYDROCHLORIDE, NV-128(Table 1). In addition, we predicted all interacting genes for drug and gene relationships (Table 2).

**Table 1:** Drug-gene interactions in the XGB model.

Search term	Match term	Gene	Drug	Interaction types	Source
Ndufab1	Ndufab1	Ndufab1	Me-344	Inhibitor	Chembl interactions
Ndufab1	Ndufab1	Ndufab1	Metformin hydrochloride	Inhibitor	Chembl interactions
Ndufab1	Ndufab1	Ndufab1	Nv-128	Inhibitor chembl	Interactions

**Table 2:** Drug-gene interactions of all intergenes.

Search term	Match term	Gene	Drug	Interaction types	Source
Ndufab1	Ndufab1	Ndufab1	Me-344	Inhibitor	Chembl interactions
Ndufab1	Ndufab1	Ndufab1	Metformin hydrochloride	Inhibitor	Chembl interactions
Ndufab1	Ndufab1	Ndufab1	Nv-128	Inhibitor	Chembl interactions
Ptbp1	Ptbp1	Ptbp1	Chembl604321	Unkonw	Dtc
Ptbp1	Ptbp1	Ptbp1	Elliptecine	Unkonw	Dtc
Ptbp1	Ptbp1	Ptbp1	Chembl586031	Unkonw	Dtc
Ptbp1	Ptbp1	Ptbp1	Chembl578502	Unkonw	Dtc
Ptbp1	Ptbp1	Ptbp1	Chembl2095095	Unkonw	Dtc
Ptbp1	Ptbp1	Ptbp1	Chembl592124	Unkonw	Dtc
Rtn4	Rtn4	Rtn4	Ozanezumab	Inhibitor|Antibody	Chembl interactions
Rtn4	Rtn4	Rtn4	Atinumab	Unkonw	Ttd
Cflar	Cflar	Cflar	Cabozantinib	Unkonw	Clearity foundation clinicaltrial
Cflar	Cflar	Cflar	Finasteride	Unkonw	Nci
Cflar	Cflar	Cflar	Bicalutamide	Unkonw	Civic
Cflar	Cflar	Cflar	Nintedanib	Unkonw	Clearity foundation clinicaltrial
Cflar	Cflar	Cflar	Dovitinib	Unkonw	Clearity foundation clinicaltrial
Cflar	Cflar	Cflar	Bay-11–7085	Unkonw	Dtc
Cflar	Cflar	Cflar	Idronoxil	Unkonw	Clearity foundation clinicaltrial
Rapgef3	Rapgef3	Rapgef3	Chembl601385	Unkonw	Dtc
Rapgef3	Rapgef3	Rapgef3	Pentabromophenol	Unkonw	Dtc
Rapgef3	Rapgef3	Rapgef3	Chembl1210769	Unkonw	Dtc
Rapgef3	Rapgef3	Rapgef3	Tanshinone I	Unkonw	Dtc
Rapgef3	Rapgef3	Rapgef3	Rafoxanide	Unkonw	Dtc
Rapgef3	Rapgef3	Rapgef3	Myricetin	Unkonw	Dtc
Rapgef3	Rapgef3	Rapgef3	Dipyridamole	Unkonw	Dtc
Rapgef3	Rapgef3	Rapgef3	Acid Blue 129 unkonw	Dtc	
Rapgef3	Rapgef3	Rapgef3	Acriflavine	Unkonw	Dtc
Rapgef3	Rapgef3	Rapgef3	Sennoside B	Unkonw	Dtc
Rapgef3	Rapgef3	Rapgef3	Chembl585591	Unkonw	Dtc
Rapgef3	Rapgef3	Rapgef3	Chembl505670	Unkonw	Dtc
Rapgef3	Rapgef3	Rapgef3	Idarubicin hydrochloride	Unkonw	Dtc
Rapgef3	Rapgef3	Rapgef3	Physodic acid	Unkonw	Dtc
Rapgef3	Rapgef3	Rapgef3	1,4-Dimethoxyanthraquinone	Unkonw	Dtc
Rapgef3	Rapgef3	Rapgef3	Gyrophoric acid	Unkonw	Dtc
Rapgef3	Rapgef3	Rapgef3	Chembl234338	Unkonw	Dtc
Rapgef3	Rapgef3	Rapgef3	Chembl1576310	Unkonw	Dtc
Rapgef3	Rapgef3	Rapgef3	2,4-Dihydroxybenzophenone	Unkonw	Dtc
Rapgef3	Rapgef3	Rapgef3	Luteolin	Unkonw	Dtc
Rapgef3	Rapgef3	Rapgef3	Daphnoretin	Unkonw	Dtc
Rapgef3	Rapgef3	Rapgef3	Chembl1256796	Unkonw	Dtc
Rapgef3	Rapgef3	Rapgef3	Chembl1306556	Unkonw	Dtc
Rapgef3	Rapgef3	Rapgef3	Hexachlorophene	Unkonw	Dtc
Rapgef3	Rapgef3	Rapgef3	Bithionoloxide	Unkonw	Dtc
Rapgef3	Rapgef3	Rapgef3	Lobaric acid	Unkonw	Dtc
Rapgef3	Rapgef3	Rapgef3	Pyrogallol red	Unkonw	Dtc
Rapgef3	Rapgef3	Rapgef3	9,10-Phenanthrenequinone	Unkonw	Dtc
Rapgef3	Rapgef3	Rapgef3	Chembl601757	Unkonw	Dtc
Rapgef3	Rapgef3	Rapgef3	Cryptotanshinone	Unkonw	Dtc
Rapgef3	Rapgef3	Rapgef3	Estropipate	Unkonw	Dtc
Rapgef3	Rapgef3	Rapgef3	Chembl515252	Unkonw	Dtc
Rapgef3	Rapgef3	Rapgef3	Purpurogallin	Unkonw	Dtc
Tubb2A	Tubb2A	Tubb2A	Vinblastine Sulfate	Inhibitor	Chembl interactions
Tubb2A	Tubb2A	Tubb2A	Eribulin Mesylate	Inhibitor	Chembl interactions
Tubb2A	Tubb2A	Tubb2A	Crolibulin	Inhibitor	Chembl interactions
Tubb2A	Tubb2A	Tubb2A	Trastuzumab Emtansine	Inhibitor	Chembl interactions
Tubb2A	Tubb2A	Tubb2A	Indibulin	Inhibitor	Chembl interactions
Tubb2A	Tubb2A	Tubb2A	Cabazitaxel	Inhibitor	Chembl interactions
Tubb2A	Tubb2A	Tubb2A	Paclitaxel	Inhibitor	Dtc|Chembl interactions
Tubb2A	Tubb2A	Tubb2A	Vinorelbine Tartrate	Inhibitor	Chembl interactions
Tubb2A	Tubb2A	Tubb2A	Colchicine	Inhibitor	Dtc|Tdgclinicaltrial|chembl interactions
Tubb2A	Tubb2A	Tubb2A	Vinflunine	Inhibitor	Chembl interactions
Tubb2A	Tubb2A	Tubb2A	Brentuximab Vedotin	Inhibitor	Chembl interactions
Tubb2A	Tubb2A	Tubb2A	Lexibulin	Inhibitor	Chembl interactions
Tubb2A	Tubb2A	Tubb2A	Ixabepilone	Inhibitor	Chembl interactions|tend
Tubb2A	Tubb2A	Tubb2A	Plinabulin	Inhibitor	Chembl interactions
Tubb2A	Tubb2A	Tubb2A	Fosbretabulin Disodium	Inhibitor	Chembl interactions
Tubb2A	Tubb2A	Tubb2A	Docetaxel	Inhibitor	Chembl interactions
Tubb2A	Tubb2A	Tubb2A	Vincristine Sulfate	Inhibitor	Chembl interactions
Tubb2A	Tubb2A	Tubb2A	Verubulin	Unkonw	Chembl interactions
Tubb2A	Tubb2A	Tubb2A	Chembl2036119	Unkonw	Dtc
Tubb2A	Tubb2A	Tubb2A	Vinblastine	Unkonw	Dtc|Tdgclinicaltrial|tend
Tubb2A	Tubb2A	Tubb2A	Podofilox	Unkonw	Dtc
Tubb2A	Tubb2A	Tubb2A	Fosbretabulin Tromethamine	Unkonw	Chembl interactions
Tubb2A	Tubb2A	Tubb2A	Chembl1935538	Unkonw	Dtc
Tubb2A	Tubb2A	Tubb2A	Maytansinol	Unkonw	Dtc
Tubb2A	Tubb2A	Tubb2A	Curcumin	Unkonw	Dtc
Tubb2A	Tubb2A	Tubb2A	Sagopilone	Unkonw	Chembl Interactions
Tubb2A	Tubb2A	Tubb2A	Vorinostat	Unkonw	Dtc
Tubb2A	Tubb2A	Tubb2A	Combretastatin A4	Unkonw	Dtc
Tubb2A	Tubb2A	Tubb2A	Maytansine	Unkonw	Dtc
Tubb2A	Tubb2A	Tubb2A	Abt-751	Unkonw	Dtc
Tubb2A	Tubb2A	Tubb2A	Paclitaxel Poliglumex	Unkonw	Chembl interactions
Tubb2A	Tubb2A	Tubb2A	Cyclostreptin	Unkonw	Dtc
Tubb2A	Tubb2A	Tubb2A	Zampanolide	Unkonw	Dtc
Tubb2A	Tubb2A	Tubb2A	Vincristine	Unkonw	Dtc|Tdgclinicaltrial|tend
Tubb2A	Tubb2A	Tubb2A	Largazole	Unkonw	Dtc
Tubb2A	Tubb2A	Tubb2A	Enmd-981693	Unkonw	Tdgclinicaltrial
Tubb2A	Tubb2A	Tubb2A	Chembl453818	Unkonw	Dtc
Tubb2A	Tubb2A	Tubb2A	Chembl2036124	Unkonw	Dtc
Tubb2A	Tubb2A	Tubb2A	Davunetide	Unkonw	Chembl interactions
Tubb2A	Tubb2A	Tubb2A	Chembl1795737	Unkonw	Dtc
Tubb2A	Tubb2A	Tubb2A	Nocodazole	Unkonw	Dtc
Tubb2A	Tubb2A	Tubb2A	Vinorelbine	Unkonw	Dtc|Tdgclinicaltrial|tend

## Discussion

AD is a neurodegenerative condition that primarily affects persons over the age of 65. It first affects memory and then progresses to permanent cognitive decline and functional disability, severely impairing the patient’s quality of life^21^. As the population ages, the rising frequency of AD imposes a tremendous financial burden on families and society^22^. Gln metabolism is gaining attention as an intriguing regulatory node that is frequently altered in a variety of pathological conditions. Gln is the most abundant non-essential amino acid in circulation, and it performs numerous metabolic functions in the cell^23^. Gln metabolism begins with the enzyme glutaminase, which deaminates it to form glutamate, an important intermediate metabolite with numerous biosynthetic applications in the cell^24^. A few recent studies have emphasized the function of GlnMgs in several aging-related illnesses. For example, Dai et al. explored the potential roles of Gln-metabolism related genes in hepatocellular carcinoma^25^. Liu et al. established a signature of Gln-metabolism for the prognosis of lung adenocarcinoma^26^. Aside from cancer, the importance of Gln-metabolism in non-cancerous diseases has received a lot of attention. Asthma, pulmonary fibrosis, and chronic obstructive pulmonary disease are a few examples. The physiological role of Gln metabolism during procession of AD is unknown. This could be an interesting line of research.

We detected 34 DEGs associated with Gln-metabolism in AD. After delving more into the role of GlnMgs in AD, we determined the Gln metabolism DEGs through intersected DEGs, WGCNA, and GlnMgs. Furthermore, four hub GlnMgs (ATP5H, NDUFAB1, PFN2, and SPHKAP) were found using LASSO regression, and their diagnostic capacity was verified using external datasets, suggesting that these genes may be involved in the AD process. The results of the aforementioned genes suggest some directions for future investigation, however there is no convincing evidence that they would be involved in the production of specific transcription factors associated with Gln metabolism control. such as CCR5, CEBPB, and CD33^27–29^. is insufficient, necessitating additional inquiry.

This ATP5H/KCTD2 locus is important in mitochondrial energy generation and neuronal hyperpolarization during cellular stress conditions such as hypoxia or glucose deprivation^30^. The risk of AD can be observed by observing the level of ATP5H/KCTD2. Panagiotis Giannos identified NDUFAB1 as a key gene in Altered mitochondrial microenvironment^31^. In acute myeloid leukemia, hypermethylation of the SKIP gene (SPHKAP) promoter silences its expression (AML). According to Essam A Ghazaly’s findings, SKIP down-regulation in AML lowers SK activity and ceramide levels, which eventually limits apoptosis in leukemia cells^32^. Chen et al. developed a Molecular Signatures of Mitochondrial Attachments database. The alteration of mitochondrial complexes that support AD onset is mediated by molecular markers implicated in oxidative phosphorylation and retrograde endocannabinoid signaling (NDUFAB1) pathways, according to this study^33^. These studies reinforce the validity and plausibility of our discoveries since these Gln metabolism DEGs seemed to be associated to the malignancy process in AD individuals. The GSE63060 analysis found that a Gln metabolism related characteristic might be used as an efficient predictive indicator. However, only a few investigations on the gene alterations associated with Gln metabolism have been conducted.

The GSVA showed the GlnMgs DEGs were enriched in primary immunodeficiency, notch and b cell receptor signaling pathway, galactose metabolism. Notch signaling pathway is highly conserved evolutionarily and is involved in the regulation of cell differentiation, proliferation and apoptosis. Notch signaling expression persists throughout the adult brain and mature differentiated cells, and is one of the major regulators of neural stem cells and neural development in the brain^34^. AD is a degenerative disease of the central nervous system. Extracellular senile plaques formed by amyloid beta deposition are one of the characteristic pathological changes of AD. Notch protein coexpresses with presenilin protein (PSs) and interacts physiologically and functionally in adult brain neurons at anaphase of mitosis^35^. Recent studies have shown that PS1 expression of AD in neural stem cells impaired the regeneration function of neural stem cells and decreased -secretase mediated proteolysis of Notch pathway, indicating that inhibition of Notch signaling pathway may play a direct role in neurodegeneration in AD^36^. Changes in brain energy metabolism have been proposed to be crucial in the development of AD. Acutely separated cerebral cortex and hippocampal slices from 3-month-old APPswe/PSEN1dE9 and wild-type control mice were incubated in conditions containing [U-13C]glucose, [1,2-13C]acetate, or [U-13C] Gln in the Andersen research. The rate of ATP production in isolated whole-brain mitochondria of these mice was reduced, and many cerebral metabolic abnormalities, including altered glucose metabolism, impaired Gln processing, and mitochondrial dysfunctions, were seen in animals prior to amyloid plaque development^37,38^. These studies show that the Notch signaling pathway and Gln metabolism play an important role in AD. However, more research into the crosstalk between these four GlnMgs in AD is required.

According to research, the particular entrance of peripheral cells into the brain parenchyma induced by damage, as well as the imbalance of the immunological milieu in the brain, are strongly associated to the development of AD. Because acquiring data within the brain is challenging, it is critical to apply machine learning algorithms to determine the link between peripheral and intracerebral data and their effect on the development of AD. Recently, neuroinflammation has been identified as one of the causes of unsuccessful therapy^39^. After an injury, neuroinflammation develops as a result of an imbalance in the synthesis and release of pro-inflammatory and anti-inflammatory cytokines from central or peripheral sources^40^. The activation of microglia is the most obvious element of neuroinflammation. Under physiological settings, activation of microglia leads in the creation of nutritional and anti-inflammatory molecules. Microglia become too active in pathological settings such as chronic stress or infection, resulting in not only increased levels of inflammatory chemicals in the brain, but also neuronal damage and death^41^. In order to successfully treat neuroinflammation and restore neurotropism and neurotransmitter function, fundamental and clinical AD research must develop unique diagnostic biomarkers from the standpoint of nerve-immunity interaction. As a result of our previous findings, we also discussed the expression of GlnMgs in the immune microenvironment. The finding show that NK cells resting, Macrophages M1, T cells CD4 naive and T cells regulatory (Tregs) were highly expressed in cluster 1. Plasma cells and Dendritic cells activated were highly expressed in cluster 2. which also proved that the pathogenesis of GlnMgs in AD is also closely related to inflammation and immune response.

The relationship between metabolism and AD has been marginally explored. Currently, some papers have used bioinfor- matics analysis to show a relationship between metabolism and AD^42–44^. Zhang et al. identified five effective biomarkers by constructing a model of age-related genes. ZNF384: A Potential Therapeutic Target for Psoriasis and AD Through Inflammation and Metabolism. Gu et al. constructed a prediction model related to iron death and performed drug prediction. They ended up with 520 effective iron metabolism-related genes. However, there are almost no studies on Gln metabolism and AD. Our study based on the mechanism of brain energy metabolism provides a reference for determining the effective brain energy metabolism in the treatment of AD. Despite this, there are currently few predictive models for GlnMgs and AD. When compared to other studies, the approach used in this study is novel. First, the current study expanded on earlier research by utilizing more GlnMgs data from the continuously updated GEO database. Second, GSE132903 were used as the primary analysis, with GSE63060 being incorporated into the common pattern for model validation. The GO and KEGG analyses, as well as the GSEA analysis, all added credibility to the study. Finally, there is almost no prediction model for GlnMgs that gives specific recommendations for future metabolic research or therapy based on metabolic interference AD. Although this study provides some context, it also has certain limitations.

Although providing theoretical underpinnings and research concepts, it has several limits. To begin, the data utilized in the study is derived from the GEO database, which makes determining the quality and trustworthiness of the statistical data challenging. Therefore, in order to increase the quality and credibility of statistical data. We chose GSE132903 was used as the main body and GSE63060 was used to verify the model with good grouping. Second, while the study focuses on mRNAs linked with Gln metabolism, the underlying processes are unknown. There is a lack of understanding of the underlying systems. As a result, there is a lack of understanding of the underlying mechanisms at work. This is one of the key limits on what we can do in vivo and in vitro. Yet, there is a dearth of understanding of the underlying processes involved. There is a lack of understanding of the underlying systems. Both in vivo and in vitro testing is an excellent idea. But so far, this provides us with other research avenues for further investigation. Additionally, the association between prognostic genes and Gln metabolism is unclear, which might provide light on the role of GlnMgs in AD. Moreover, there are few external data sources available to verify the model’s veracity, making validation difficult. The following are the study’s difficulties. This risk model is heavily reliant on publicly available databases. Also, protein expression may differ from RNA expression, demanding more study with a larger data set.

## Conclusions

AD occurs and progresses as a result of interactions between multiple targets, pathways, signaling pathways, and mech- anisms, and the regulatory process is synergistic and bi-directional. GlnMgs affects the production of ATP5H, NDUFAB1, PFN2, and SPHKAP, which can activate or inhibit the notch signaling pathway, primary immunodeficiency, B cell receptor signal pathway.a˘Notch and other signaling pathways regulate immune-inflammatory responses, neuron remodeling, and other processes, as well as the onset and progression of AD.

This work has significant limitations, despite the fact that it presents some theoretical underpinnings and research proposals for GlnMgs in AD. The following ideas for future enhancement are made: (1) Because the current data is generated from the GEO, determining the trustworthiness and quality of the statistical data is difficult. In the future, the number of data sources will be increased while the data offset will be reduced. (2) More scientific and clinical studies will be conducted to determine whether effective therapies can enhance the neurological and immune functions of AD patients by regulating these GlnMgs in the brain.

## Materials and methods

### Raw data processing

GEO was searched for mRNA expression. Series: GSE132903 and GSE63060. Platform: GPL10558 and GPL6947 (Table 1). GSE132903 and GSE63060 were used as the trian and test groups respectively. Strategy for searching (’Alzheimer’s disease’ [MeSH] mRNA [All Fields] and normal) AND (’Homo sapiens’ [Organism] AND ’Non-coding RNA profiling by array’ [Filter]). MSigDB was used to retrieve 26 GlnMgs (Table S1).

### Analysis of DEGs

Perl (https://github.com/Perl) matched and sorted transcription data to acquire exact mRNA data. The IDs were converted into gene names. After the data standardization of GSE132903 using the normalize Between Arrays function in the limma package, PCA was conducted by using the factoextra package. The Differentially expressed genes (DEGs) between AD and non-demented controls (ND) were analyzed. The DEGs were screened with the criteria of |Fold2FC|> 1 and p < 0.05. To show significantly deregulated genes, a heat map was created using ggplot2 and the "ComplexHeatmap" package.

### Immune cell infiltration

The immune cell components in adipose tissue were analyzed via CIBERSORT. We built barplot and corplot with the limma package to show the results of immune cells.

### Cluster analysis

We used the Limma and ConsensusClusterPlus package to do cluster analysis. With the clustering variable (k) at 2, a strongest intragroup correlation and a weakest intergroup correlation were observed. The GlnMgs associated with prognosis were classified into cluster 1 and 2. We also performed a consensusScore based on this result^45^. The limma was utilized to discover changes in particular genes between subtypes and tissue types^46^.

### Enrichment analysis

Using Gene Ontology (GO) and Kyoto Encyclopedia of Genes and Genomes (KEGG), the biological function and path- ways linked with the DEGs were then investigated. The biological pathways associated with the DEGs were then examined using Gene Ontology (GO). Biological processes (BP), molecular functions (MF), and cellular components (CC) controlled by the differentially expressed GlnMgs were further investigated using R. “c5.go.bp.v7.5.1.symbols” gene sets were obtained from MSigDB. GSVA was used to compute the process score by “GSVA” package.

### Co-expression gene identification

WGCNA is an algorithm for clustering genes into distinct modules and determining the correlations between modules and disease features. To examine the genetic processes implicated in the pathogenesis of AD, the “WGCNA” package was used to build a co-expression network. The co-expression network was built utilizing just the genes with the highest 25% variance from GSE132903. The dynamic cutting tree approach was used to combine modules with a threshold of 0.25. Other criteria were utilized to build the co-expression network, including: soft threshold power () based on the scale-free topology requirement (an independence value of R^2^ = 0.85) by using the select Soft Threshold function; and minimum genes of each module = 30. Pearson correlation analysis was used to identify possible relationships between modules and patient clinical variables.

### GlnMgs identification

To find the GlnMgs, we intersected the DEGs from major modules (WGCNA), Gln, and cluster hubGenes. A Vnnmap was used to visualize the overlapping genes. As previously stated, biological processes and enrichment routes were also uncovered. We separated the GSE132903 into training cohorts after identifying Hub GlnMgs. The “glmnet” package was used to discover the hub DEGs, with the smallest lambda defined as the best value. In each sample, the DEGs predicting score was computed. The diagnostic and discriminative utility of GlnMgs in AD and ND was evaluated using receiver operating characteristic curve analysis. The external validation dataset was GSE63060.

### Drug-gene interactions

With the advancement of bioinformatics, the construction of biological models and the identification of efficient biomark- ers has become more significant in the diagnosis and prevention of clinical disorders. Even if the biomarkers are established, the crucial issue is determining how to use them in the clinic. As a result, medication prediction based on successful indicators will be critical in the future prevention and treatment of AD. Validated biomarkers provide some reference for clinical treat- ment. Therefore effective drug prediction is very important. We used the DGIdb database (https://dgidb.genome.wustl.edu/) to make drug predictions for both the obtained hub genes and so the intersection gene in the XGB model.

### Ethics approval and consent to participation

This manuscript is not a clinical trial, hence the ethics approval and consent to participation are not applicable.

## Supplementary Information

Supplementary Tables.

## Data Availability

The datasets generated during and/or analyzed during the current study are available in the appendix. For data citations of datasets uploaded to e.g. figshare, please use the howpublished option in the bib entry to specify the platform and the link, as in the Hao:gidmaps:2014 example in the sample bibliography file.

## Abbreviations

AD: Alzheimers disease
GO: Gene ontology
TCM: Traditional Chinese medicine
MF: Molecular functions
KEGG: Kyoto encyclopedia of genes and genomes
GEO: Gene expression omnibus
Gln: Glutamine
BP: Biological processes
CC: Cellular components
DEGs: Differentially expressed genes
