# Supplementary material for: A novel Alzheimer’s disease prognostic signature: identification and analysis of glutamine metabolism genes in immunogenicity and immunotherapy efficacy
Source: Sci Rep. 2023 Apr 27;13:6895. doi: 10.1038/s41598-023-33277-x (PMC10140060; doi:10.1038/s41598-023-33277-x)
Supplement: Supplementary file 1 — Supplementary Tables. [file 41598_2023_33277_MOESM1_ESM.doc]

A Novel Alzheimer's Disease Prognostic Signature: Identification and Analysis of Glutamine Metabolism Genes in Immunogenicity and Immunotherapy Efficacy

**Supplementary appendix to the manuscript**

Contents of supplementary appendix

[Appendix 1 3](#__RefHeading___Toc3582)

[Datasets and Glutamine Metabolism 3](#__RefHeading___Toc9952)

[Table 1. Glutamine metabolism genes 3](#__RefHeading___Toc30109)

[Appendix 2 4](#__RefHeading___Toc12397)

[DEGs linked to glutamine metabolism geness 4](#__RefHeading___Toc29016)

[Table 3. 17 DEGs linked to glutamine metabolism genes. 4](#__RefHeading___Toc26419)

[Appendix 3 5](#__RefHeading___Toc30138)

[Table 3. chromosomal positions of Gln metabolism-related genes. 5](#__RefHeading___Toc27665)

[Appendix 4 6](#__RefHeading___Toc3257)

[Table 4. Co-expression Network Construction and Module Detection. 6](#__RefHeading___Toc9064)

[Appendix 5 7](#__RefHeading___Toc10835)

[Co-expression Network Construction and Module Detection of Clustering 7](#__RefHeading___Toc21981)

[Table 5. Co-expression Network Construction and Module Detection of Clustering. 7](#__RefHeading___Toc28363)

[Appendix 6 14](#__RefHeading___Toc31760)

[Table 7a. InterGenes. 14](#__RefHeading___Toc3074)

[Appendix 7 15](#__RefHeading___Toc13590)

[Table 7. ImportanceGene.XGB. 15](#__RefHeading___Toc28302)

# Appendix 1

**Datasets and Glutamine Metabolism**

**Table 1. Glutamine metabolism genes**

| GLYATL1B | ASL | GMPS | GLUD1 |
| --- | --- | --- | --- |
| CPS1 | ASNS | ARHGAP11B | GLUL |
| CTPS1 | LGSN | GLYATL1 | CAD |
| SIRT4 | PFAS | GFPT2 | SLC38A1 |
| PHGDH | ASNSD1 | NR1H4 | CTPS2 |
| GFPT1 | PPAT | MECP2 | NIT2 |
| GLS2 | GLS |  |  |

# Appendix 2

## DEGs linked to glutamine metabolism genes

**Table 2. 17 DEGs linked to glutamine metabolism genes**.

| ID | GSM3895951 | GSM3895952 | GSM3895953 | GSM3895954 | GSM3895955 |
| --- | --- | --- | --- | --- | --- |
| PHGDH | 11.180241 | 11.539428 | 12.010266 | 11.059456 | 10.74979 |
| GFPT1 | 7.266225 | 6.804652 | 7.194715 | 7.509814 | 8.032237 |
| GLS2 | 6.927243 | 6.364165 | 6.9786 | 7.048373 | 7.210731 |
| GLS | 11.49108 | 10.356343 | 11.329176 | 11.61743 | 11.36666 |
| GLUL | 6.22626 | 6.23758 | 6.218962 | 6.213229 | 6.236183 |
| MECP2 | 6.368501 | 6.295154 | 6.378554 | 6.821752 | 6.714383 |
| ASL | 6.491535 | 6.403115 | 6.479733 | 6.480271 | 6.47398 |
| ASNS | 9.494435 | 9.094216 | 10.220714 | 9.675237 | 9.759263 |
| LGSN | 6.183825 | 6.085287 | 6.181745 | 6.060044 | 6.119547 |
| PFAS | 8.238216 | 7.3401 | 8.131499 | 8.411373 | 8.42562 |
| ASNSD1 | 8.721838 | 8.434012 | 9.043284 | 9.292732 | 9.706556 |
| PPAT | 7.926515 | 7.779399 | 8.074837 | 8.056741 | 8.041824 |
| CTPS2 | 6.899524 | 6.689522 | 6.778198 | 6.860797 | 6.787153 |
| NIT2 | 7.219728 | 6.865237 | 6.996298 | 7.589607 | 7.493045 |
| ARHGAP11B | 6.335472 | 6.24958 | 6.251264 | 6.162422 | 6.209143 |
| GLYATL1 | 6.352728 | 6.372357 | 6.325746 | 6.239311 | 6.279044 |
| NR1H4 | 6.247357 | 6.346381 | 6.290426 | 6.325279 | 6.274166 |

# Appendix 3

**Chromosomal positions of Gln metabolism-related genes**

**Table 3. chromosomal positions of Gln metabolism-related genes**.

| Chromosome | chromStart | chromEnd | Gene |
| --- | --- | --- | --- |
| chr1 | 40979335 | 41012565 | CTPS1 |
| chr1 | 119659798 | 119744215 | PHGDH |
| chr1 | 182381704 | 182392206 | GLUL |
| chr2 | 27217390 | 27243943 | CAD |
| chr2 | 69319769 | 69387254 | GFPT1 |
| chr2 | 189661385 | 189670831 | ASNSD1 |
| chr2 | 190880827 | 190965552 | GLS |
| chr2 | 210477682 | 210679107 | CPS1 |
| chr3 | 100334701 | 100361635 | NIT2 |
| chr3 | 155870536 | 155944026 | GMPS |
| chr4 | 56393362 | 56435615 | PPAT |
| chr5 | 180300690 | 180353387 | GFPT2 |
| chr6 | 63275951 | 63319977 | LGSN |
| chr7 | 66075798 | 66093558 | ASL |
| chr7 | 97852118 | 97872542 | ASNS |
| chr10 | 87050486 | 87094866 | GLUD1 |
| chr11 | 58905398 | 59043527 | GLYATL1 |
| chr12 | 46183063 | 46270017 | SLC38A1 |
| chr12 | 56470944 | 56488414 | GLS2 |
| chr12 | 100473708 | 100564413 | NR1H4 |
| chr12 | 120302316 | 120313249 | SIRT4 |
| chr15 | 30624494 | 30772993 | ARHGAP11B |
| chr17 | 8247618 | 8270491 | PFAS |
| chrX | 16588003 | 16712936 | CTPS2 |
| chrX | 154021573 | 154137103 | MECP2 |

# Appendix 4

**Co-expression Network Construction and Module Detection**

**Table 4. Co-expression Network Construction and Module Detection**.

| LCMT1 | CHD7 | C15orf52 | SLITRK1 | TMEM150A | TRABD |
| --- | --- | --- | --- | --- | --- |
| PRKX | WASF1 | SLC25A18 | SLC16A14 | NME3 | PDGFRB |
| C6orf168 | PTBP1 | VIL2 | FAM65C | ATP5L | NRN1 |
| AHNAK | ZHX3 | RAPGEF3 | CISD1 | INPP5D | SLC15A3 |
| WWC3 | SRGAP1 | PTRF | LAMA5 | LASS1 | EZR |
| STK38 | ITPRIP | PLXNB1 | FBXW4 | KRT222 | ADCYAP1 |
| TNS1 | PEX11B | MKL2 | ATP6V1E1 | CALD1 | MRPS30 |
| BNIP3 | MAP3K6 | ITPKB | TMEM216 | GSDMD | PFN2 |
| LOC100134011 | HSPB3 | NAP1L5 | NDUFAB1 | TEAD2 | LOC728014 |
| ATP5H | SPHKAP | TUBB2A | NINJ1 | RTN4 | ITSN1 |
| C5orf22 | MLLT6 | PCSK1 | CTDSP2 | CFLAR | NOTCH1 |
| ZNF184 | CCKBR | NPTX2 | KIAA1881 | NFKBIA | FYCO1 |

**Appendix 5**

**Co-expression Network Construction and Module Detection of Clustering**

**Table 5. Co-expression Network Construction and Module Detection of Clustering**.

| AKT1 | RIC3 | FAM135B | CLDN5 | DRG1 | C6orf142 |
| --- | --- | --- | --- | --- | --- |
| TMEM207 | HMGCLL1 | ITFG1 | BEX4 | FEZ2 | P2RY11 |
| RNASEH2C | FOXJ2 | PIK3CB | LOC642377 | ZNF650 | TCEAL1 |
| NHLRC3 | ANKRD35 | ZHX3 | LOC341457 | CTPS | NOP14 |
| TOX3 | ALS2 | TMEM219 | BEX5 | TTC37 | PM20D1 |
| PHF19 | TIMM23 | RAB6A | RTN1 | ERI3 | EXTL2 |
| MYADM | CHN1 | ATP2A2 | FLJ38717 | LOC642357 | TANC2 |
| LOC651075 | TNFSF14 | LOC389523 | TMEM155 | NRXN1 | UBE2T |
| MAT2B | SCUBE2 | CDC40 | CBLN4 | FLJ35258 | DDX1 |
| DGKI | TM2D3 | KLHDC2 | SFMBT2 | ZNF791 | RALGAPA1 |
| LOC647150 | MRLC2 | FZD3 | DDX27 | RASAL3 | PREP |
| GLT8D2 | HSF2 | C9orf114 | SCN2B | RPS6KC1 | STEAP2 |
| UCP3 | DHX36 | SLC44A2 | CPNE4 | APPBP2 | SLITRK1 |
| ARSK | HEATR5B | CADM4 | SNCA | LOC440145 | ZSWIM4 |
| RAD51C | GGCT | FAM134B | PRKCB1 | LMNA | CLCN7 |
| HS6ST2 | STARD3 | BEND6 | LPPR4 | DNAJC19 | VASH1 |
| AKAP11 | TPD52L2 | C1orf128 | ITPR1 | C18orf8 | LOC643466 |
| DSCAM | LRRC14 | RAPGEF2 | DCLK1 | B4GALT4 | SPNS1 |
| LOC643384 | RDBP | DNM1L | TUBB2A | MED22 | ATP5B |
| LGMN | DDX25 | REEP5 | EPB41L3 | WDR37 | PHLPP2 |
| MMS19L | ASB1 | PIGQ | TAGLN3 | REV1 | MBLAC2 |
| BMPER | LOC728661 | LCAT | PNMAL1 | SLC25A10 | ZFAND5 |
| ACYP1 | YTHDF2 | HN1 | PNMA2 | LFNG | PLEKHO2 |
| JAK2 | FUK | STARD8 | TSPYL1 | FKBP8 | ID2 |
| C14orf173 | CCND3 | PTH1R | PREPL | KIAA0146 | RG9MTD1 |
| C6orf66 | FLJ20699 | TMEM35 | ZCCHC12 | LOC646808 | SLC16A14 |
| ARL2BP | TRIM37 | LOC100129064 | YWHAG | C11orf48 | GPC6 |
| SNORA58 | COX6C | MGC27121 | ELMOD1 | GFPT1 | DCAF6 |
| MEX3A | PGAP1 | U2AF1L2 | LOC339879 | N4BP2L1 | PRR14 |
| SERPINB6 | TOR1A | CITED2 | KIAA1107 | AQP11 | RAMP1 |
| TBC1D19 | PFKFB4 | LOC100133163 | NELL1 | RAB3GAP2 | ARMCX1 |
| SLC6A10P | CLTA | TXLNA | SCG2 | INSIG2 | NUDT16 |
| LRRC40 | SCPEP1 | GPRASP2 | GNG2 | TSNAX | CTDSPL |
| ACSL4 | DNAJC12 | LOC100133600 | EPHA4 | COPA | FLJ22795 |
| PJA1 | ZNF184 | PSMG1 | RGS7 | SMYD2 | MOBKL2A |
| ZNF766 | FIZ1 | RND2 | C1orf173 | SNORD104 | PPP2CA |
| RDH14 | TSPAN9 | THOC2 | GLRB | CLRN1 | KIAA1468 |
| TXNDC16 | KBTBD6 | CIB2 | MAL2 | USP15 | C9orf64 |
| MOV10 | SLC6A15 | ZNF827 | HPRT1 | CCDC104 | WASL |
| CSTF3 | CRTC3 | SLC9A3R1 | NAP1L3 | C14orf104 | CAPRIN2 |
| PPP4R4 | FAM125A | NPEPL1 | DIRAS2 | IARS | NRCAM |
| DYRK1A | ZMYM4 | PAM | UCHL1 | SHMT2 | NTNG1 |
| SLC19A2 | B4GALT5 | WHAMM | DYNC1I1 | SLC25A29 | ABCE1 |
| ZNF331 | SLC6A8 | ANKRD46 | CAP2 | BNIP3 | NELF |
| FOSL2 | ATP6V1H | TBPL1 | SERPINI1 | PUM2 | ZNF296 |
| NGLY1 | EIF4E3 | STXBP5L | TSPAN13 | B3GALNT1 | RRBP1 |
| TBK1 | PRMT6 | TMEM222 | EFCBP1 | GOPC | EIF4A2 |
| PDCD6IP | ACTN2 | C7orf70 | NEFM | MRPL3 | SLC39A10 |
| SYDE2 | PHTF1 | HOXB6 | NELL2 | EIF2C2 | SATB2 |
| C5orf44 | NUPL2 | SEC61A1 | CHGB | CBLN2 | ACACB |
| GAS2L3 | PFN2 | LOC401152 | RGS4 | PIP5K2A | MORF4L2 |
| VAMP4 | FTO | CLDND2 | SYT1 | IRX1 | MAP2K1 |
| ADCK4 | COX5A | PMPCB | RAB13 | CENPB | RIMBP2 |
| UBE2F | CHL1 | C20orf55 | LOC100132532 | JAZF1 | DLC1 |
| DHX37 | ARID3B | PEX11B | PPIAL4A | VASN | GOT1 |
| LOC650840 | ZBTB46 | WRB | EML3 | FLJ46906 | DACH2 |
| KLHL7 | PODN | GPR22 | FAM3C | TRABD | TMEM156 |
| RTKN | CD68 | MOBKL3 | ATP6AP2 | XK | UBQLN2 |
| ARAP1 | DYNLT3 | MAP3K6 | PRKCB | LLPH | LOC100128002 |
| LOC650116 | RPL13L | POU2F1 | PGRMC1 | LOC134997 | KIAA1279 |
| MIR300 | C4orf41 | LZTFL1 | RAPGEF3 | RTN4 | LOC220686 |
| UBXN1 | IFI35 | LUZP2 | PRNP | VDAC1 | TMEM91 |
| LOC341965 | YWHAZ | NR1H3 | TBC1D9 | CFLAR | KPNA3 |
| CAPRIN1 | RNF38 | PCMT1 | PTRF | BTBD10 | DMXL2 |
| EEF1E1 | VPS37B | SPHKAP | ZNF786 | OLFM3 | BBS4 |
| EFCAB7 | KLHL9 | LDHA | ZCCHC24 | PNMA1 | SLC25A46 |
| RPS6KB2 | VTA1 | ABCB7 | FBXO34 | INPPL1 | TRIM56 |
| ASPH | ACSF2 | ASAP3 | CUEDC1 | C15orf52 | LRP3 |
| CKAP5 | NME3 | LOC648980 | ZCCHC6 | RICS | C12orf51 |
| LOC440731 | SUSD1 | IL4R | PLXNB1 | UBXN2A | WFS1 |
| HCCS | LRRC46 | C9orf37 | TMEM14A | ATP1A1 | IFI27L2 |
| WDR21A | ZDHHC17 | TSGA14 | OXR1 | TOMM20 | IPO5 |
| SGK3 | MDH2 | FIBCD1 | FAM19A2 | DKFZp434K191 | RAB11FIP4 |
| DIS3L2 | DERL1 | TSPAN33 | MOAP1 | GABARAPL1 | CNNM3 |
| FAM175B | C12orf76 | OSBPL10 | CDH13 | UBE2N | MAP3K10 |
| RPAIN | ARMCX5 | LSM11 | LRP11 | GPR137C | STAU2 |
| QPCT | NIF3L1 | C20orf94 | AMAC1L3 | SYNJ2BP | SLBP |
| NT5C3 | ARHGAP4 | ISCA1 | OMG | SLC25A18 | DPY19L1 |
| ALX1 | RAB22A | MLLT6 | COX19 | SLC25A4 | SNX22 |
| TXN2 | MFN2 | C21orf2 | NCOA7 | LOC727948 | LOC729389 |
| ZNF434 | GSPT2 | NOV | TNFRSF10C | GRIN2C | MAPK9 |
| PGBD1 | STXBP5 | TM9SF2 | C6orf117 | LOC653158 | FBXW4 |
| EFCAB4A | PRPS1 | SDK2 | RAXL1 | XAF1 | CDO1 |
| FASTKD5 | C9orf5 | ANKMY2 | C14orf174 | VIL2 | DCXR |
| PSMA4 | UBE2M | SCHIP1 | C9orf130 | STAMBPL1 | GLCE |
| MTERFD1 | LANCL2 | RRAGA | SC5DL | DNM3 | OPA1 |
| ZNF688 | C12orf4 | ZDHHC23 | AASDHPPT | OAT | HECTD1 |
| CTDSP1 | WDSUB1 | ZNF787 | BEX2 | HCG9 | LOC100134266 |
| PIGB | BAP1 | NAV3 | NAP1L2 | VPS35 | SIX5 |
| CLIP2 | RWDD2A | CMAS | GABRA5 | GOLSYN | RRAGB |
| C5orf42 | SEPX1 | LOC728014 | B3GNT6 | TSPAN7 | MYO9B |
| URM1 | ACPL2 | LOC100132774 | TMEM137 | NECAP1 | TCTEX1D2 |
| NY-REN-7 | CHMP1A | NCRNA00085 | LRFN5 | BST2 | STAM |
| ZDHHC21 | ZNF526 | C2orf30 | NEK6 | SCOC | CDH10 |
| RANBP1 | HADHA | MRPS30 | TMOD1 | ACTR10 | GOLT1B |
| SNTG1 | OGT | EFEMP2 | ANKRD33 | KIAA1467 | CSAD |
| LZTS2 | UBA5 | MAPK6 | LONRF2 | LRCH4 | TADA1L |
| BRPF1 | GPR137 | ERCC5 | HDAC6 | PI4KAP2 | LMBR1 |
| LOC100132266 | AKAP6 | LOC647340 | LRRC4C | LYL1 | AGK |
| NAT5 | C6orf120 | TSSC4 | AUH | LANCL1 | GPM6A |
| OCRL | SMYD3 | LOC646786 | LOC653505 | UQCRC2 | BMPR2 |
| JAGN1 | SLC35A1 | RHOC | NDUFAB1 | DOCK3 | SNRPN |
| SEC24D | ZNF385B | LOC100133478 | SLC25A39 | PFTK1 | PAFAH1B1 |
| LOC100132213 | LOC647328 | C2orf32 | C1orf149 | TMX3 | PTBP1 |
| SIGIRR | APP | LASS1 | LOC727967 | PPP3CA | LOC648399 |
| EFNA4 | SNHG9 | TUBA1A | LOC283267 | KIAA0367 | MIR2116 |
| SMAP1 | ATP5L | SLC27A1 | TM2D2 | PCDH17 | PDDC1 |
| ADAM17 | FCRLB | LIME1 | PRDM9 | PIAS4 | MTCH1 |
| CD151 | PLEKHA4 | ZNF462 | MAP3K11 | SLC7A5 | KIAA0430 |
| GPR19 | MGC29506 | OR2T11 | PACSIN3 | PCDH20 | KCNN3 |
| ORC4L | PLEKHH3 | CSRNP2 | TP53BP1 | HERC1 | FLJ42709 |
| LOC728431 | FHL1 | SFXN5 | ISCU | ABHD7 | ESRRAP2 |
| LOC642082 | CD200 | DNCL1 | PPP1R14B | LOC100131277 | BBC3 |
| UBE3C | PSMC2 | LOC100130511 | ZNF672 | YPEL5 | COX6B1 |
| AVPR2 | LOC653073 | NPTN | APOO | LOC729081 | ARL6IP5 |
| C10orf137 | C3orf14 | ATP6V1D | HOXD3 | RNY1 | SRPK1 |
| LOC100129522 | ARL6IP1 | ARAF | GPT | EZR | CD79A |
| CLK3 | C9orf125 | NR2F1 | CPNE8 | ZNF25 | C3orf31 |
| PRDM10 | EHD2 | NEU4 | NAPEPLD | LPCAT3 | ARID4B |
| YTHDC2 | NOTCH4 | TSPYL5 | ARHGAP20 | EID2 | CD82 |
| MBIP | CUL2 | SETX | SLC24A6 | RGS7BP | KLHL12 |
| CCDC126 | CYP11A1 | LOC196752 | LOC653344 | CNTN3 | CCNH |
| HIVEP1 | HIST1H1A | SUB1 | PLEKHO1 | PCLO | FAM92A1 |
| PIGN | IER3IP1 | RASD1 | RTCD1 | CSPG4 | LOC730820 |
| PCSK4 | EBNA1BP2 | PPP1R1A | ROM1 | NBEA | SFRS3 |
| IFIT1 | C13orf37 | UCHL5 | TUSC3 | ATP6V1G2 | MTMR4 |
| PDCD2L | TMEM66 | C1orf59 | C14orf2 | NDE1 | FAM129B |
| RAB11A | C22orf13 | LOC100133888 | TTC38 | FXYD5 | FASTKD2 |
| SFRS13B | ARV1 | ZC3H15 | PTDSS1 | FAM38A | NUDT21 |
| C12orf11 | MTPN | MUM1L1 | EIF5B | FAM102B | MTX2 |
| TGFB1I1 | SFRS2 | C3orf51 | LOC643433 | SLC25A12 | EPS15 |
| MAN1C1 | ZNF444 | LOC92017 | LOC648149 | LOC654155 | MAP3K3 |
| EVI5L | FHOD3 | RPN2 | FAM3A | SCN3B | MIR1185-1 |
| REPS1 | LOC644694 | ARGLU1 | UBA7 | GLRX | COPS3 |
| ORMDL3 | ZBTB42 | NSF | DPM1 | KRCC1 | MKRN2 |
| DGCR6 | ACTR6 | GPR37L1 | FAM83F | TCEAL6 | CREBL1 |
| RBM11 | WDR7 | CD47 | YWHAB | BAI3 | LOC731751 |
| TTC23 | INHBB | MIDN | LOC439953 | LOC400446 | KIAA1539 |
| ACVR2A | SLC29A4 | TP53I13 | BHLHB9 | EIF1B | MUC1 |
| DGCR11 | PDHX | MAPKAPK3 | KITLG | DNAJB2 | BTBD3 |
| FHL2 | CXADR | LOC100134134 | NDUFV2 | SNX6 | HOMER3 |
| DTX1 | ST6GALNAC6 | TBL1X | NDUFS5 | THNSL1 | LOC729142 |
| LOC643206 | FBXO3 | HSFYP1 | C14orf93 | RBBP5 | HERC3 |
| MUTYH | DGCR6L | NDFIP2 | NDUFS7 | YEATS2 | CALM2 |
| FAM53B | ARL1 | CALD1 | TRIM41 | LOC653421 | SERBP1 |
| SMPDL3A | C1orf69 | SPC24 | MAGEL2 | LOC92659 | THYN1 |
| PLXND1 | RASSF4 | MYOM1 | DENND4C | ALS2CR4 | IMPAD1 |
| CDC42SE2 | MGAT1 | HLF | DPYSL2 | OPN3 | ATP5H |
| IGDCC3 | LOC390671 | SGIP1 | RGL3 | TFB2M | FGF23 |
| COQ10B | LOC649447 | CHST6 | C12orf43 | PDIK1L | MRPL32 |
| DFFA | TMEM120A | MXD4 | LOC645321 | FBXO28 | TMEM115 |
| LOC100130623 | YARS2 | LOC100131541 | ZNF295 | LOC646135 | C5orf22 |
| POMGNT1 | C5 | GSDMD | AP1G1 | LRMP | LOC100128528 |
| SNORA8 | TTC19 | FLJ12078 | TTC33 | LOC653567 | C2orf47 |
| WDR23 | ZSCAN18 | KHSRP | ZBTB7A | GNL1 | SEH1L |
| LRP5 | ADCY4 | TMC6 | SC65 | SP1 | ANKRD13C |
| MRPS21 | LIMS2 | KIAA1881 | KPNA1 | DGAT1 | UBQLN1 |
| FLJ20628 | MAP2K7 | RANBP6 | OR9A4 | TNIP1 | HCCA2 |
| IMP3 | PRRG2 | LOC90624 | SLC4A1AP |  |  |

# Appendix 6

**InterGenes**

**Table 6. InterGenes**.

| BNIP3 | FBXW4 | CALD1 | RAPGEF3 | SLITRK1 | MRPS30 |
| --- | --- | --- | --- | --- | --- |
| ATP5H | PTBP1 | GSDMD | PTRF | SLC16A14 | LASS1 |
| C5orf22 | ZHX3 | KIAA1881 | PLXNB1 | NDUFAB1 | ATP5L |
| ZNF184 | PEX11B | EZR | TUBB2A | LOC728014 | MLLT6 |
| PFN2 | MAP3K6 | TRABD | SLC25A18 | C15orf52 | CFLAR |
| NME3 | SPHKAP | RTN4 | VIL2 |  |  |

# Appendix 7

**ImportanceGene.XGB**

**Table 7.** ImportanceGene.XGB.

| variable | permutation | dropout_loss | label |
| --- | --- | --- | --- |
| Type | 0 | 0.441644736725318 | XGB |
| ATP5H | 0 | 0.44164852551823 | XGB |
| NDUFAB1 | 0 | 0.441739104538682 | XGB |
| PFN2 | 0 | 0.443298945174563 | XGB |
| SPHKAP | 0 | 0.443751492549397 | XGB |
